# Regulation of dietary intake of protein and lipid by nurse-age adult worker honeybees

**DOI:** 10.1242/jeb.230615

**Published:** 2021-02-08

**Authors:** Daniel Stabler, Mushtaq Al-Esawy, Jennifer A. Chennells, Giorgia Perri, Alexandria Robinson, Geraldine A. Wright

**Affiliations:** 1 Department of Zoology, University of Oxford, Oxford OX1 3SZ, UK; 2 Faculty of Medical Sciences, Newcastle University, Newcastle upon Tyne NE1 7RU, UK; 3 Department of Plant Protection, Faculty of Agriculture, University of Kufa, PO Box 21, Najaf, Iraq

**Keywords:** Bee, Nutrient regulation, Essential amino acids, Protein, Lipid, Geometric framework

## Abstract

Essential macronutrients are critical to the fitness and survival of animals. Many studies have shown that animals regulate the amount of protein and carbohydrate they eat for optimal performance. Regulation of dietary fat is important but less often studied. Honeybees collect and consume floral pollen to obtain protein and fat but how they achieve the optimal balance of these two macronutrients is presently unknown. Here, using chemically defined diets composed of essential amino acids and lipids (lecithin), we show that adult worker honeybees actively regulate their intake of lipids around optimal values relative to the amount of protein in their diet. We found that broodless, nurse-age worker honeybees consume foods to achieve a ratio between 1:2 and 1:3 for essential amino acids to lipid or ∼1.25:1 protein to fat. Bees fed diets relatively high in fat gained abdominal fat and had enlarged hypopharyngeal glands. In most cases, eating diets high in fat did not result in increased mortality. Importantly, we also discovered that the total quantity of food the bees ate increased when they were given a choice of two diets relatively high in fat, implying that dietary fat influences bee nutritional state in a way that, in turn, influences behaviour. We speculate that dietary fat plays a critical role in maintaining workers in the nurse-like behavioural state independently of the influence of queen pheromone.

## Abstract (precis)

**Summary:** Nurse-age adult worker honeybees regulate their intake of dietary essential amino acids and fat to a ratio of 1:2. Honeybees gain weight when fed diets high in fat.

## INTRODUCTION

Protein and fat are essential macronutrients required in most animals' diets. Protein provides essential amino acids whereas oils or fats provide the essential fatty acids linoleic acid and α-linolenic acid. To satisfy their nutritional needs for growth, maintenance and reproduction, animals have evolved mechanisms for the detection and regulation of the procurement of these nutrients. The regulation of macronutrient intake has been formalized in a modelling framework called the geometric framework (GF) for nutrition. The GF is designed to measure the strategies animals employ when regulating two types of essential nutrients simultaneously (Simpson and Simpson, 1990; Raubenheimer and Simpson, 1993). It specifically employs the use of diets of specific ratios of macronutrients. Animals are given one of two types of conditions: they are either confined to a diet of a defined ratio or they are given access to at least two different foods of specific ratios of two macronutrients. Using chemically defined diets in this way makes it possible to study the trade-offs made when animals are confined to eat substandard diets. If an animal is given a choice of two diets (i.e. a ‘paired diet’ design), it is possible to identify how animals self-select a presumed optimal quantity of each macronutrient through the measurement of the consumption of both diets.

Most research employing the GF has focused on how animals acquire carbohydrates and protein, in part because these nutrients are required in the largest amounts by herbivores. A few insightful studies employing the GF in predatory arthropods have revealed that dietary lipids are also actively regulated (Jensen et al., 2011, 2012; Mayntz et al., 2005). For example, predatory ground beetles regulate their intake of protein relative to lipid in a way that suggests both nutrients are costly when overeaten (Jensen et al., 2012). Of the two nutrients, lipids were more strongly regulated than protein (Jensen et al., 2012). Egg production in ground beetles is also optimized around a 2:1 protein to lipid ratio (Jensen et al., 2012). The extent to which protein and lipid intake is regulated, however, depends on the species in question. The orb web spider, *Argiope keyserlingi*, does not appear to actively regulate its intake of protein to lipid, although the composition of the food does affect its own body composition (Hawley et al., 2014).

Most plant material has very little fat (<1%; Ivanova et al., 2012). For this reason, herbivorous insects might be fat-limited in the diet, especially if they require the essential fatty acids α-linolenic and linoleic acid. Animals assimilate fat in their bodies through multiple mechanisms. Non-essential fatty acids can be created from excess carbohydrate (Arrese and Soulages, 2010) or provided by gut endosymbionts (Temara et al., 1991; Pranal et al., 1996; Douglas, 1998, 2009). Bees and other pollinators acquire dietary protein, lipid and carbohydrate (starch) from floral pollen (Wright et al., 2018). Floral pollen is similar to insect prey in its composition, as it is largely made up of protein and lipid (Roulston and Cane, 2000; Human and Nicolson, 2006). Foraging-age bumblebees (*Bombus* *terrestris* and *Bombus* *impatiens*) regulate their intake of protein and lipid to ratios between 14:1 and 12:1 when fed holidic diets (14:1 for *B. impatiens* and 12:1 for *B. terrestris*) (Vaudo et al., 2016). The regulation of fat, therefore, may be easier to study in animals adapted to a diet high in fat (Wilder et al., 2013).

To date, few studies have tested macronutrient regulation in eusocial insects and these have focused mainly on protein and carbohydrates. Eusocial workers in ants, honeybees and bumblebees actively regulate their intake of protein [or essential amino acids (EAAs)] because it is costly; over-ingestion increases the risk of mortality for ants, honeybees and bumblebees (Altaye, et al., 2010; Pirk et al., 2010; Dussutour and Simpson, 2012; Paoli et al., 2014; Stabler et al., 2015). Additionally, the regulation of macronutrients by adult workers depends on their caste and age. For example, nurse-age adult worker honeybees regulate their intake of protein (P) and carbohydrate (C) to a ratio of 1:50 P:C when they are fed with diets composed of sugar and EAAs; however, as they age, their requirement for dietary carbohydrate increases relative to EAAs such that their optimum approaches a ratio of 1:250 P:C (Paoli et al., 2014). Likewise, foraging-age adult worker bumblebees (*Bombus terrestris*) regulate their intake of P:C to 1:149 when fed with diets composed of protein (casein) and carbohydrate (sucrose). However, when fed with diets composed of free amino acids, they alter their intake towards a carbohydrate-biased ratio of 1:560 EAA:C (Stabler et al., 2015).

Eusocial bees collect and store pollen for use by the whole colony. The quantities of protein and fat in floral pollen vary greatly depending on floral origin. Protein ranges between 2 and 60% and lipid between 2 and 20% of pollen (Roulston and Cane, 2000). A study of bee-pollinated plant species' pollen reported protein to lipid (P:L) ranges of 13:1 to 1:5 P:L (Vaudo et al., 2020). In a honeybee colony, the nurse-age bees consume pollen (stored as bee bread, pollen mixed with honey) and create glandular secretions such as royal jelly to provision developing larvae with sufficient nutrition to reach the stage of pupation. A reasonable hypothesis would be that nurse-age honeybees, the main consumers of bee bread and pollen, regulate their intake of fats relative to protein in proportions that are similar to royal jelly they produce (i.e. ∼2:1, see Wright et al., 2018), but this has never been tested. Here, we have performed a series of experiments using a chemically defined diet to identify the optimal protein and fat requirements of nurse-age adult worker honeybees. These experiments were designed to test how adult worker honeybees regulate their intake of essential amino acids relative to lipid (EAA:L). To identify the costs of consuming non-optimal ratios of EAA:L, honeybees were confined to a diet made of a specific ratio of EAA:L. To identify the range of ratios of EAA:L that were optimal for nurse bees, honeybees had a choice of foods containing a specific ratio of EAA:L versus a food made of EAAs without lipids or they were given a choice of two foods with different ratios of EAA:L.

## MATERIALS AND METHODS

### Animals

Frames of sealed brood were collected from 6–10 (depending on the experiment) honeybee (*Apis mellifera carnica* Pollman 1879) colonies kept at Newcastle University, UK. Brood frames were suspended in ventilated boxes and kept in an incubator at 34°C and 60% relative humidity (RH). Newly emerged female workers were brushed from the frames daily and combined to form a mixed population in order to give a stronger representation of a population of bees across the treatment groups (Paoli et al., 2014). Cohorts of ∼30 bees from the mixed population were then housed in ventilated Perspex feeding cages (11×6×20 cm). Feeding cages had 6 holes for feeding tubes, with 3 holes positioned at each end of the cage. Feeding tubes were modified 2 ml microcentrifuge tubes with 4×2 mm holes drilled along their length. The number of feeding tubes provided was experiment dependent and any blank holes were blocked with strong tape. Liquid diets were added to the feeding tubes, and the feeding cages with bees were placed in an incubator at 34°C and 60% RH. Bees were housed in feeding cages for 10 days and any dead bees were counted and removed daily.

### Chemically defined diets

Diets were designed so that the ratio between total EAA:L could be varied using liquid diets as in Vaudo et al. (2016). Three experiments were designed to test how honeybees regulate the consumption of essential amino acids and lipid. Diets were composed of the 10 EAAs, sucrose and lipid (see Vaudo et al., 2016). Amino acids were present at equimolar concentrations and each amino acid contributed 0.004 mol l^−1^ to make a final ‘total’ amino acid concentration of 0.04 mol l^−1^ (as in Paoli et al., 2014). Reagent grade L-amino acids were sourced from Alfa Aesar. Sucrose was provided at 1 mol l^−1^, making an EAA to carbohydrate molar ratio of 1:25 EAA:C (Table S1); this ratio was chosen based on previous work showing the intake targets achieved by newly emerged bees using the same diets (Paoli et al., 2014). The EAA:C ratio was constant in all diets, but the EAA:lipid (EAA:L) ratio depended on the treatment group. The concentration of EAA in the diet was 0.6% of the solution. The lipid source was soy lecithin (Optima Health & Nutrition, Bradford, UK) as in Vaudo et al. (2016). Consumption of diets was measured by weighing feeding tubes prior to placing them in the feeding cages and then again after 24 h. Tubes were weighed and replaced every 24 h for 10 days for all experiments. To control for potential changes in tube weight because of water loss from evaporation, control boxes without bees were run in parallel. Daily consumption of each diet was then adjusted for its specific evaporation value.

To relate the EAAs in diet to a specific amount of protein, we also conducted a series of control experiments. Casein is often used as the protein source in nutrition studies with insects and has been used with bees (Altaye et al., 2010; Pirk et al., 2010; Stabler et al., 2015). Casein does not have the EAAs in the same proportions of our equimolar diet. To generalize our data to a ‘real world’ situation where bees were eating protein, we tested if the ratio of EAAs in our ‘equimolar’ diet influenced the quantity of food eaten by honeybees. To do this, we compared their food consumption with that of bees fed a diet of EAAs at the proportions found in casein. Bees had access to two feeding tubes. One diet contained amino acids in 1 mol l^−1^ sucrose and the second contained 1 mol l^−1^ sucrose only. One cohort of bees was fed an equimolar EAA diet and a second cohort was fed the amino acid profile of casein (Table S2). Bees ate 3.9 times more AAs when feeding on casein AAs compared with an equimolar diet (Table S2). When combined with data from bumblebees, this suggests that the proportion of EAAs in the diet can influence the total amount of EAA eaten. We use these data to infer the quantity of a ‘protein’ that might be consumed when bees eat pollen (Stabler et al., 2015).

### Experiment 1. Confined treatments

The first experiment designed was a confined assay in which bees had access to one treatment solution containing EAAs, lipids and sucrose and a second solution containing only 1 mol l^−1^ sucrose. EAA and C were held constant in all treatment diets but the ratio of EAA:L was modified for 8 treatment diets by varying lipid content. The treatments were modified to include 1:0, 25:1, 10:1, 5:1, 1:1, 1:5, 1:10 and 1:12.5 EAA:L (Table S1). Each cohort of 30 bees had access to one of the specified EAA:L treatments and 1 mol l^−1^ sucrose (*N*=10 cohorts of 30 bees per treatment).

### Experiment 2. Amino acid-paired treatments

The next experiment was designed to identify the range of values for the EAA:L ratio that we identified as a putative intake target in experiment 1. We achieved this by giving honeybees access to three diets: (1) one EAA:L treatment diet (25:1, 10:1, 5:1, 1:1, 1:5, 1:10 or 1:12.5); (2) a 1:25 EAA:C diet (no lipid); (3) and a 1 mol l^−1^ sucrose diet. The concentration of the EAA and carbohydrate components of diet were held constant, and lipid was varied as in experiment 1 (*N*=10 cohorts of 30 bees per treatment).

### Experiment 3. Lipid-paired treatments

In this experiment, cohorts of honeybees were fed with two diets that differed in their EAA:L ratio. The logic of this experiment was to test whether bees regulated their intake of EAA:L to a specific ratio (Raubenheimer and Simpson, 1999). We used information from experiments 1 and 2 to design the treatments, as we predicted that the intake target for EAA:L was between 1:1 and 1:5 EAA:L. The nutrient space was limited such that all treatments were given one high lipid diet (1:5 EAA:L) and an additional diet of one of the following: 25:1, 10:1, 5:1 and 1:1. As in experiment 1, all diets contained 1 mol l^−1^ sucrose with an EAA:C ratio of 1:25 (M/M). A separate 1 mol l^−1^ sucrose diet was also provided (*N*=10 cohorts of 30 bees per treatment).

### Hypopharyngeal gland size

To assess how EAA:L diet affects hypopharyngeal gland development (HPG), the size of hypopharyngeal glands was measured from a subsample of bees from the confined treatments experiment. To measure HPG size, the heads of 10 bees from each treatment were dissected. A needle was used to remove the dorsal plane of the head, and HPGs were removed with fine forceps. After being removed from the head, HPGs were added to 50 µl phosphate buffered saline (PBS). As an indicator of HPG size, the diameter of the shorter axis of 10 neighbouring acini per gland were measured under a microscope (Leica M125, Leica) and the average value for each bee was used as a unit of replication (Lass and Crailsheim, 1996; Babendreier et al., 2005). Images were taken of each acini and ImageJ software (v.1.51j8) (Schneider et al., 2012) was used to measure the diameter against a reference scale (Corby-Harris and Snyder, 2018).

### Abdominal fat content

Ten bees from each cohort of the confined treatments (experiment 1) and amino acid-paired treatments (experiment 2) experiments were used to measure abdominal fat content. Bees were frozen at −80°C at the end of each experiment and were then dissected on ice. The abdomen was removed from the body and the gut was dissected out while the bee was frozen to prevent any fat in the gut contaminating the sample. A glass scintillation vial was pre-weighed, and the abdomen samples were added to it. Vials containing abdomens were placed in an oven at 60°C and samples were dried to constant mass. Once dry weight was recorded, 5 ml petroleum ether was added to each vial and the lid was replaced. Samples were left for 5 days at room temperature and the petroleum ether was removed and the samples were then dried to constant mass. Fat content was recorded as the difference in pre and post petroleum ether extraction weight (Ellers, 1995; Strohm, 2000; O'Neill et al., 2011).

### Statistical analyses

Analyses were carried out using IBM SPSS v.24 (IBM Corp. Released 2016. IBM SPSS Statistics for Macintosh, Version 24.0. Armonk, NY: IBM Corp). Average daily volume consumption was compared using 2-way analysis of variance (ANOVA) with treatment and solution as main effects and with Šidák's *post hoc* tests for multiple comparisons. The intake of each nutrient was calculated (mg) and presented as intake per bee. Consumption of nutrients (mg) was compared using multiple analysis of variance (MANOVA) with total EAA, lipid and carbohydrate intake as dependent variables. Intake targets were identified using Šidák *post hoc* tests. To assess the differences in daily consumption of nutrients, repeated measures ANOVA was applied to daily consumption values of EAA, carbohydrate and lipid, with EAA:L treatment as the main effect. Survival data were analysed using Cox regression (Cox proportional hazards model). In the confined assay, the 1:0 (no lipid) diet was used as the indicator variable. Survival in the protein-paired and lipid-paired assays were also analysed with Cox regression using the most dilute lipid diet as the indicator variable. The effect of EAA:L treatment on abdominal fat content was compared using a one-way ANOVA. HPG acini diameter was applied as the dependent variable in a one-way ANOVA with EAA:L treatment as the main effect.

## RESULTS

### Adult worker honeybees compromise their intake of EAA when diet is too lipid-rich

**Fig. 1.**
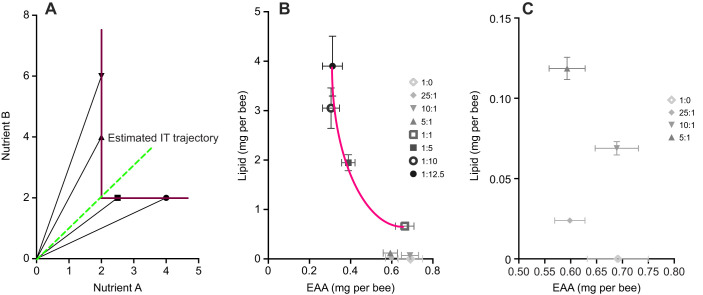
**Honeybees adopt a rule of compromise when confined to** **essential amino acid (EAA) and lipid diets.** (A) Schematic of predicted behavioural adjustment when animals are confined to diets outside of their optimal range of two nutrients (or intake target, IT). Data points further along the *x*-axis indicate that nutrient A is being over ingested to maintain a minimum requirement of nutrient B. Similarly, as data points move further along the *y*-axis, the animal over-ingests nutrient B to maintain a minimum intake of nutrient A. A diet close to the optimum would result in lower overall consumption, and so fewer compensations would need to be made. The estimated optimum in this case, would follow the trajectory of the green line. (B) Bees adopt a rule of compromise when confined to diets ranging between 1:1 and 1:12.5 EAA:L. The switch in consumption of nutrients between 1:1 and 1:5, suggests that the optimum ratio of EAA:L lies within this range. (C) Replot of lower EAA:L treatments. Honeybees are not able to consume enough dietary lipid in treatments less concentrated than 5:1 EAA:L. In these cases, their consumption of lipid is not regulated relative to EAA. The data in A and B are representative of the same animals. Error bars indicate s.e.m.; *N*=10 cohorts of 30 bees per EAA:L treatment.

When bees were confined to a diet with a specific ratio of EAA:L, they regulated their intake of macronutrients in a way that suggested a ‘rule of compromise’ (Fig. 1A; Raubenheimer and Simpson, 1997). This rule of compromise was observed for only a subset of the diets (1:1–1:12.5) (Fig. 1B). For these diets, honeybees regulated their intake such that they ate no less than 0.3 mg per bee EAA (diets 1:12.5 and 1:10) and no less than 0.66 mg per bee of lipids (diets 1:1) (Table 1, Fig. 1B). The point of change in plots of this kind has been used previously to identify the putative optimal ratio of macronutrients (Raubenheimer and Simpson, 1997). In our data, the predicted optimal value for EAA:L was ∼1:2. Because they were confined to the diets, bees fed with this range of EAA:L actively adjusted their intake of EAAs; these bees consumed ∼1.9× less amino acids than the other treatments (Table 1, Fig. 1B, Šidák *post hoc*, *P*<0.001) and achieved an EAA:C ratio of 1:378 (Fig. S1A, MANOVA; Table 2, Šidák *post hoc*, *P*>0.05). The C:L ratio depended on the diet; the total amount of carbohydrates was the same, but lipid varied as a function of diet (Table 1, MANOVA; Table 2, Fig. S1B).

**Table 1.**
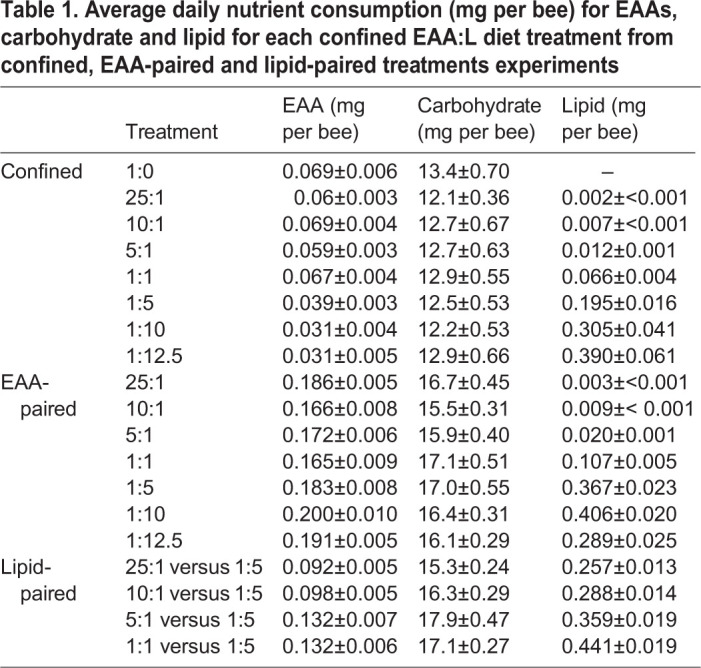
**Average daily nutrient consumption (mg per bee) for EAAs, carbohydrate and lipid for each confined EAA:L diet treatment from confined, EAA-paired and lipid-paired treatments experiments**

**Table 2.**
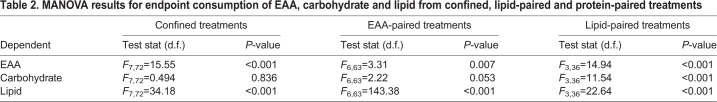
**MANOVA results for endpoint consumption of EAA, carbohydrate and lipid from confined, lipid-paired and protein-paired treatments**

When fed with diets relatively low in lipids (1:0–5:1), the bees did not exhibit the rule of compromise (Fig. 1B,C). These honeybees could not reach their intake target for EAA:L because the diets were too dilute in lipids; instead, the bees ate the diets without adjustment for lipid content (Fig. 1C). They still actively regulated their EAA:C intake (1:0, 25:1, 10:1 and 5:1), achieving a ratio of ∼1:198 EAA:C (MANOVA, Table 2, Fig. S1A, Šidák's *post hoc* tests, *P*>0.05).

**Fig. 2.**
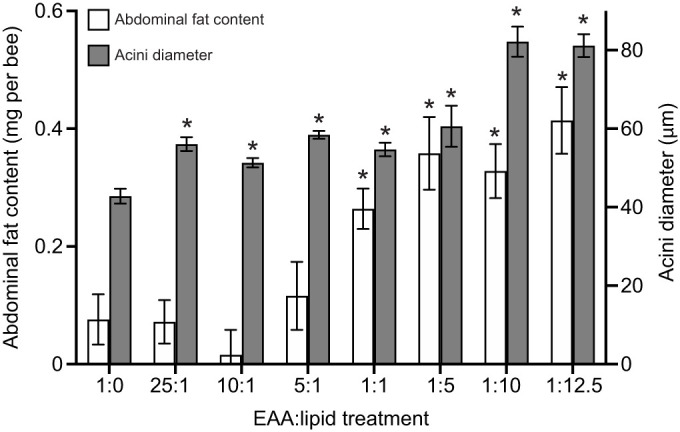
**Increased abdominal fat and HPG acini size are linked to consumption of dietary lipid.** The fat body measured in 10-day-old honeybees was elevated in diets of equal EAA:L and diets more concentrated in lipid. Diets with low lipid have similar fat body reserves as bees that ate no lipid. The presence of all concentrations of lipid increases the measured acini size of hypopharyngeal glands compared with the treatment diet without lipid. Error bars indicate s.e.m.; acini size, *N*=10; abdominal fat content, *N*=10. *P<0.05 with Šidák's *post hoc* test.

Bees confined to a high lipid EAA:L had larger hypopharyngeal gland acini than those fed with proportionally less fat (Fig. 2, ANOVA, *F*_7,32_=24.7, *P*≤0.001). Bees fed with high lipid EAA:L diets also had proportionally more body fat (Fig. 2, 1-way ANOVA, *F*_7,72_=10.1, *P*<0.001). Confining bees to specific EAA:L diets did not influence their survival (Fig. S2, Coxreg, χ_7_^2^=8.71, *P*=0.275); ∼94% of the bees in all the treatments were alive at day 10.

### Honeybees regulate their intake of EAA:L to a region between 1:2 and 1:3

**Fig. 3.**
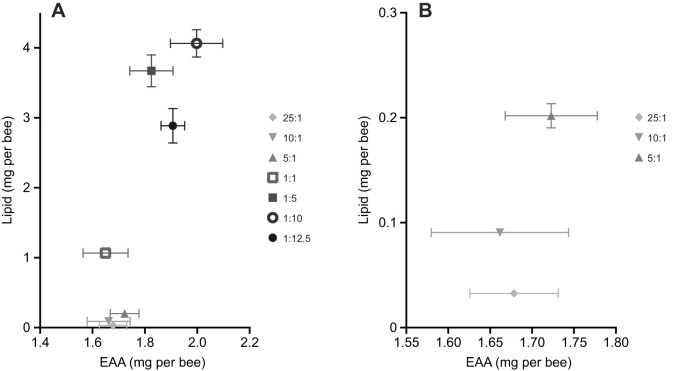
**Bees simultaneously balance their EAA to carbohydrate and EAA to lipid ratios.** (A) Cumulative EAA and lipid intake after 10 days for all dietary treatments. When concentration of lipid in diet exceeds 1:5 EAA:L, bees compensate for this by consuming less of the lipid diets (1:5, 1:10 and 1:12.5 EAA:L) and achieve an EAA:L intake between 1:1.5 and 1:2 EAA:L. (B) Dilute lipid diets replotted. When lipid in diet is low, bees do not balance the nutrient intake and consume EAA:L ratios close to those predicted of random feeding. Error bars represent s.e.m.; *N*=10 cohorts of 30 bees per EAA:L paired treatment.

To verify the predicted optimal intake target from the confined treatments experiments, we designed an experiment in which bees could freely regulate their intake of carbohydrate and EAA but were restricted to a specific ratio of EAA:L. This allowed the bees to independently regulate their intake of EAA:C. Honeybees consumed the diets in a way that depended on the EAA:L ratio (Fig. 3A,B, MANOVA; Table 2, carbohydrate *F*_6,63_=2.22, *P=*0.053, EAA *F*_6,63_=3.31, *P=*0.007, lipid *F*_6,63_=143, *P*<0.001; Fig. S3). They also regulated their intake of EAA:C in a relatively narrow range of values (1:82–1:104) (Table 1, Table 3, Fig. S3A).

**Table 3.**
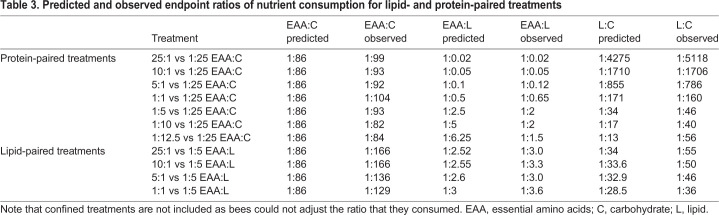
**Predicted and observed endpoint ratios of nutrient consumption for lipid- and protein-paired treatments**

As before, when bees were confined to a specific EAA:L ratio, honeybees fed with diets in the range between 1:1 and 1:12.5 EAA:L exhibited active regulation of the EAA:L (Fig. 3A, Table 3). However, the EAA:L ratio achieved depended on the diet (Table 1, Table 3). Honeybees fed with the 1:5 and 1:10 EAA:L diets were able to regulate their intake so that they maintained a ratio of 1:2 of EAA:L (Table 3, Fig. 3A; Šidák's *post hoc* EAA *P*=0.905, carbohydrate *P*=0.999l, lipid *P*=0.76; Fig. S3A,B). Bees in the 1:12.5 diet treatment achieved a ratio of 1:1.4 EAA:L by reducing their intake of the EAA:L diet and consuming proportionally more EAA (Table 1, Table 3, Fig. 3). The bees in the relatively high lipid diets regulated their intake of L:C within a range of 1:40–1:160 (Table 3, Fig. S3B).

When bees were provided with a diet with more EAAs than 1:1 EAA:L (5:1, 10:1 and 25:1), the EAA:L ratio achieved was very near to a predicted EAA:L if the bees ate from the tubes without active regulation of lipids (Table 3). These bees regulated their intake of EAA:C along a narrow range from 1:92–1:99, indicating active regulation of these two macronutrients (Table 3). The ratio of L:C, however, varied over a much wider range from 1:786 to 1:1706 and was near to the ratio predicted by random feeding (Table 3).

**Fig. 4.**
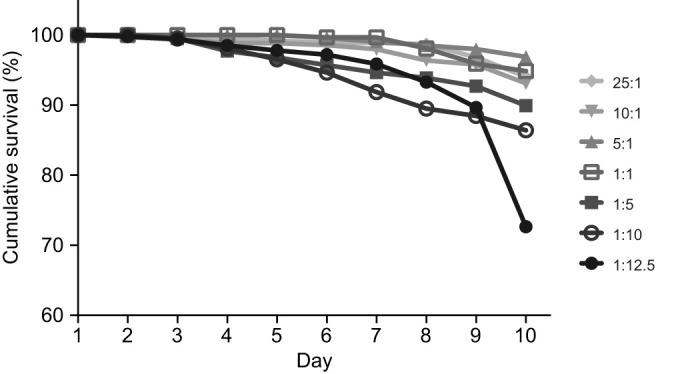
**Bees fed with the highest concentrations of lipid had greater risk of mortality than bees feeding on lower lipid diets.** Bees on the 1:10 and 1:12.5 EAA:L treatments are 2.2- and 4.8-times, respectively, more likely to die over the course of the experiment than bees on the lowest lipid diet (25:1 EAA:L). *N*=10 cohorts of 30 bees per EAA:L paired treatment.

The bees fed with the relatively higher lipid ratios exhibited a higher risk of mortality (Fig. 4, Coxreg, 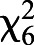
=27.64, *P*<0.001). In fact, bees feeding on 1:10 treatment had 1.85× greater risk of mortality over 10 days than bees on the most dilute lipid diet (25:1) (*P=*0.019). Bees feeding on all other diets had similar risk of mortality (Fig. 4).

**Fig. 5.**
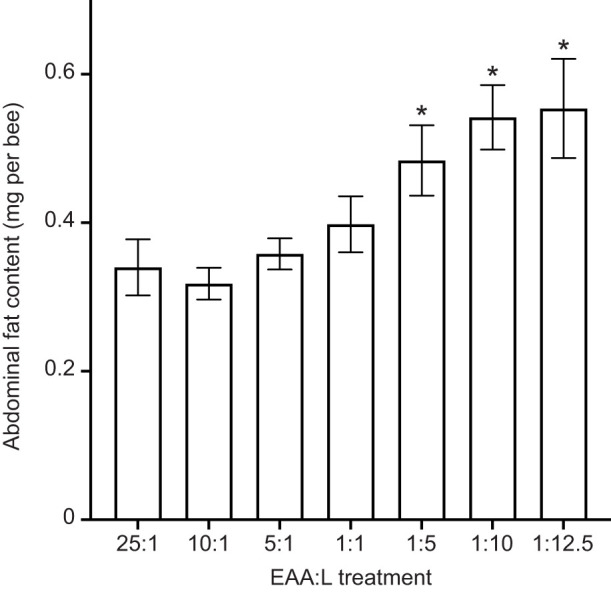
**Abdominal fat content depends on sufficient dietary lipid.** When bees feed from higher EAA:L diets (1:5 to 1:12.5) paired with EAA:C diets, their abdominal fat content is significantly greater than with lower lipid diets. Error bars represent s.e.m.; *N*=10.

The abdominal fat content of bees from the amino acid-paired treatments experiment was also influenced by EAA:L treatment (Fig. 5, 1-way ANOVA, *F*_6,63_=5.45, *P*<0.010). Bees fed with EAA:L ratios of 1:5, 1:10 and 1:12.5 had significantly more abdominal fat than all the other EAA:L treatments (LSD *post hoc* tests, *P*≤0.038).

### Honeybees fed with proportionally higher fat diets consume more food

**Fig. 6.**
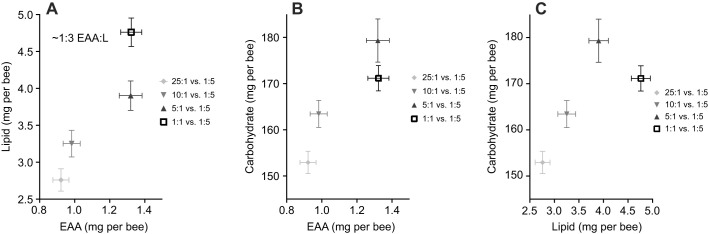
**High lipid diets increase honeybee feeding.** (A) After 10 days of feeding, bees balance their EAA:L intake to ∼1:3. (B) When diets are high in lipid, the EAA:C intake target is skewed from 1:166 EAA:C in the 25:1 and 10:1 treatments, towards increased consumption of EAAS (∼1:130 EAA:C). (C) L:C ratios skew from carbohydrate towards greater lipid, as the lipid content of the treatment diet increases. Error bars represent s.e.m.; *N*=10 cohorts of 30 bees per EAA:L paired treatment.

**Fig. 7.**
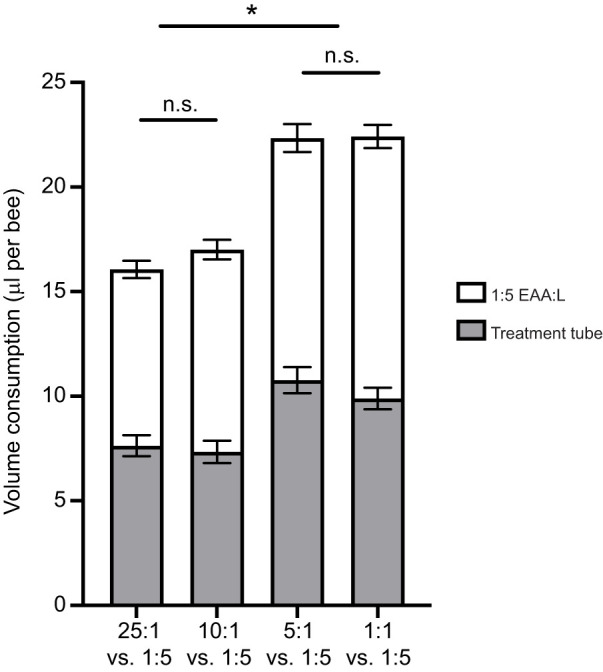
**When lipid in diet is high, honeybees eat more.** When both diet pairs are relatively high in lipid (5:1 versus 1:5 and 1:1 versus 1:5), bees eat significantly more than when lipid pairs are relatively low (25:1 versus 1:5 and 10:1 versus 1:5). Error bars represent s.e.m.; *N*=10 cohorts of 30 bees per EAA:L paired treatment. *P<0.05 with Šidák's *post hoc* test; n.s. denotes no significant difference.

The previous experiments indicated that bees regulated their intake towards a ratio of ∼1:2 EAA:L. We performed one final experiment to test whether bees could indeed regulate to a predicted intake target when given a choice of two diets containing different ratios of EAA:L. Unexpectedly, the EAA:L treatment pair significantly influenced the total quantity of food consumed over the course of the experiment (Fig. 6, Fig. 7, Table 1, MANOVA; Table 2, two-way ANOVA, treatment×solution, *F*_6,108_=5.114, *P*<0.001). Bees provided with the more concentrated EAA:L diets consumed more total food overall (5:1+1:5 and 1:1+1:5) than bees fed the other two diet pairs (Figs 6 and 7, Table 1, LSD *post hoc P*≤0.014).

The bees in 3 out of 4 treatments (25:1+1:5, 10:1+1:5, and 5:1+1:5) regulated their intake to ∼1:3 EAA:L (Fig. 6A, Table 3, Šidák *post hoc*, all *P>*0.17). Bees in the highest lipid diet pair (1:1+1:5) consumed the most lipid, with a ratio of 1:3.6 EAA:L (Fig. 6A, Šidák *post hoc P*≤0.012). Bees feeding on diet pairs where both treatments were relatively concentrated in lipids (5:1+1:5 and 1:1+1:5) consumed ∼1.4× more EAA resulting in EAA:C ratios of 1:136 and 1:129, respectively (Fig. 6B, Table 1, Table 3, Šidák *post hoc*, all *P*≤0.01). Bees feeding on diet pairs with one dilute lipid treatment (25:1+1:5 and 10:1+1:5) consumed similar amounts of EAAs and carbohydrate, achieving an IT of 1:166 EAA:C (Fig. 6B, Table 3, Šidák *post hoc*, all *P>*0.173). The range of values for the L:C ratio ranged from 1:36 to 1:55 (Fig. 6C, Table 1, Table 3). None of the diet pairs significantly affected the rate of mortality over the 10 days of the experiment (Fig. S4, Coxreg, 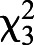 =2.33, *P=*0.51).

### Characterising relative protein from EAA consumption

When bumblebees feed on solutions containing EAA, they consume around 3 times less of the amino acid diet than they do from a diet containing protein (sodium caseinate) (Stabler et al., 2015). In order to estimate how the results found in our current study may be translated from EAA:L to P:L regulation, we carried out an experiment in which bees were provided with one of two treatment diets; one that contained equimolar essential amino acids, and a second with the amino acid profile of casein protein. In doing so we found that bees ate 3.9 times more amino acids on the casein profile treatment than on the equimolar treatment (Table S2), a similar finding to that of *B. terrestris* (Stabler et al., 2015).

## DISCUSSION

Broodless nurse-aged honeybees adjusted their intake of diets in our experiments to reach an EAA:L ratio between 1:2 and 1:3. Using an adjustment, we estimate that bees fed with protein rather than free amino acids regulate their food intake to an equivalent ratio of ∼1.25:1 P:L (see Table S2). Thus, the experiments presented here show that nurse bees without brood bias their intake of EAA:L towards fats when compared with the ratio required to produce royal jelly. Our experiments also showed that the context of the dietary choices (i.e. high lipid versus low lipid pair) affected the total amount of food eaten. When given a choice of two diets with a relatively high lipid content, honeybees increased their total food intake by 25% compared with levels measured when they had a choice among lower lipid diets (Fig. 7). Bees avoided overeating dietary lipid: when they were confined to a high lipid diet (e.g. 1:12.5), they consumed the minimum food needed to meet their requirements for EAA in order to limit lipid intake. Unlike our previous work with bumblebees, we did not observe a strong cost to overeating lipid in these experiments. There was an increased risk of mortality in one treatment diet (experiment 2, 1:10 EAA:L) but dietary lipid was not associated with increased risk of mortality in the other treatments or in any of the treatments used in experiments 1 and 3.

Our original prediction was that bees would regulate their intake of lipid relative to protein in proportions that are similar to royal jelly they produce (i.e. ∼2:1, see Wright et al., 2018). This ratio is quite similar to the measurements made of protein and lipid in larval honeybee tissue (2.4:1 P:L) (Ghosh et al., 2016). However, chemical analyses of adult bee tissues indicate that adult nurses have a P:L ratio skewed towards protein (7.4:1) (Ghosh et al., 2016). In our experiments, newly emerged bees self-select diets that are ∼1.25:1 P:L (we approximate this from our EAA:L diets, see Table S2), and thus, closer in ratio to what is required for royal jelly production. A previous study that used a mixture of honeybee-collected pollen reported that newly emerged honeybees did not exhibit a preference for diets based on their P:L content (Corby-Harris et al., 2018). However, the range of diets provided to these bees excluded the values that we identified as optimal, and, therefore, were likely to have been too narrow to draw this conclusion. If the bees had fed randomly in the experiments of Corby-Harris et al. (2018), they would have achieved P:L intakes in a narrow range between 3:1 and 4.5:1. Our methods permitted us to explore a much larger ‘nutritional space’, making it possible to identify the range of values they actively self-select.

A foraging honeybee colony will collect pollen from a diverse range of flowering plant species at any given time, depending on the local environment. Pollen nutrient composition varies considerably among plant species (Roulston and Cane, 2000). The P:L ratio in floral pollen has a large range; in some cases, lipid content surpasses protein levels (Roulston and Cane, 2000; Vaudo et al., 2020). In a honeybee colony, the foraging honeybees do not consume floral pollen, they only collect it; the nurse-age bees eat the pollen and make glandular secretions to feed larvae and the queen. A recent analysis of the protein and lipid of honeybee-collected pollen identified that they collect pollen with an average P:L composition of 1.5:1 (Vaudo et al., 2020). This ratio, however, is quite different to the reported ratios of honeybee-collected pollen from a study in Israel (Avni et al., 2014). These authors found that foraging honeybees collect pollen from 22–94% of the flowering plants available, depending on location (Avni et al., 2009); in Israel, the plant species' pollen had an average P:L ratio of ∼10:1 (Avni et al., 2014). It is likely, therefore, that honeybee colonies must adapt to the local forage and that nurse bees must balance their intake of P:L over the range of possible values in floral pollen. This could be a direct result of the whole colony's nutritional state, but no studies have examined this to date.

When honeybees collect pollen, it is mixed with nectar and stored in cells in the colony. Stored pollen (bee bread) comes from several different floral origins. Bee bread, therefore, creates a nutrient profile for P:L that is less varied than that of individual plant species (10–30% protein and 3–8% lipid; Wright et al., 2018). Using these values, at its greatest extremes, the P:L ratios of bee bread vary from 10:1 to 1.25:1. Interestingly, these values fall across the range predicted by the studies of Vaudo et al. (2020) and Avni et al. (2014).

Bumblebees, by contrast, appear to require diets much higher in protein than in fat. In a previous experiment, we measured the regulation of P:L of two species of bumblebee (*B. impatiens* and *B. terrestris*) (Vaudo et al., 2016). Foragers of these species regulate their intake of P:L to between 12:1 and 14:1 on their own, but to a ratio of 3:1 when they are foraging for a whole colony (Kraus et al., 2019). In addition, the P:L ratio of mixed plant species pollen collected by *B. impatiens* reportedly has a ratio near to 4:1 P:L (Vaudo et al., 2020). These ratios are considerably less concentrated in fat than we find for broodless, nurse-aged honeybees; however, bumblebees do not produce royal jelly as honeybees do and so their requirement for dietary lipid may be less. A closer examination of the P:L ratios found in the tissues of different castes in bumblebee colonies may reveal that the ratio of P:L required depends on bumblebee caste and sex.

Although we did not investigate it here, the reproductive and behavioural castes in a honeybee colony are likely to have different dietary needs for protein and lipid. It is also possible that our estimation of the preferred P:L ratio was biased more towards lipid because we worked with bees in a queenless, broodless setting in the lab. When bees are in a colony setting, they are exposed to many other in-hive factors that can influence their behaviour. Here, we have removed these stimuli in order to explore the nutrient balancing behaviour of the adult worker in a controlled setting (Paoli et al., 2014; Stabler et al., 2015; Vaudo et al., 2016). We expect that the EAA:L regulation reported here indicates the requirements of individual adult workers and that this may be different to when they are exposed to brood and queen pheromones. In our previous work, for example, we know that nurse-age honeybees regulate their intake of EAA:C towards diets that are considerably more concentrated in EAA than the ratio selected by foragers (Paoli et al., 2014). Thus, the differences in the P:L ratios identified as optimal for nurse-age honeybees in our study and in our previous work with bumblebees is likely due both to age and species differences in the relative demand for protein and lipid. We predict that when honeybees are consuming food to rear brood, they need proportionally more fat in their diet. This is because nurse-age honeybees produce glandular secretions (e.g. royal jelly) that have an estimated P:L ratio of 2:1 (Howe et al., 1985; Wright et al., 2018).

The present experiments and our previous work indicate that honeybees have a greater cost to over- or under-ingesting protein than fat. The main purpose of eating protein for a sterile adult worker is to acquire the essential amino acids needed for somatic maintenance and growth and to produce glandular secretions to feed to brood and the queen (De Groot, 1953; Crailsheim, 1990). Imbalance of protein intake increases the risk of mortality for nurse- and foraging-aged bees (Paoli et al., 2014). There are also other sublethal consequences, including impaired royal jelly production, facilitated through reduced HPG development, varying degrees of ovarian activation (increased on higher quality proteins) and by the level of mobilised protein and amino acids found in the haemolymph, depending on the protein source (Altaye et al., 2010; Stabler et al., 2015). Although we show here clear behavioural regulation of dietary lipid, the consequences of imbalanced P:L intake are less severe than the imbalanced intake of protein or carbohydrate. In our experiments, nurse-aged bees could consume diets as high as 1:12.5 EAA:L and still survive. The main consequence of diets high in fat for nurse honeybees is that they exhibit an increase in body fat.

Body fat acquired in diet could affect the progression of honeybees from nurse-age to forager. Previous work has shown that queen mandibular pheromone (QMP) plays an important role in controlling the behavioural caste trajectories of workers in the colony (Pankiw, 2004; Fischer and Grozinger, 2008). In the presence of the queen, nurse-age bees remain nurse-like (Jay, 1970, 1972; Jay and Jay, 1976; Kaatz et al., 1992). When they are removed from the influence of QMP, they begin to transform from nurse to forager (Pankiw et al., 1998). This transition to forager is accompanied by radical changes in their physiology that include the loss of body fat and reduced HPG size (Crailsheim, 1986, 1992; Toth and Robinson, 2005; Fischer and Grozinger, 2008) and is found in other social hymenopteran insects (Blanchard et al., 2000; Toth and Robinson, 2005).

Although pheromones are important in this behavioural caste transition, bees that cannot acquire fat from diet become foragers precociously (Toth et al., 2005). These experiments indicate that body fat plays a role in the regulation of state in honeybees. Our data support this and show that fat in diet also influences how much food bees eat. When the diet choices we gave to bees were relatively high in fat, bees ate more food and also changed the ratio of P:L towards more lipid. Fat in diet is likely to stimulate the production of brood food, just as absence of fat accelerates the transition to the forager caste. When bees are starved of pollen, and so limited in protein or lipid, ecdysteroid hormone production increases in correlation with depletion of the fat body (Corby-Harris et al., 2019). Furthermore, when bees are injected with ecdysterone makisterone A, their hypopharyngeal glands decrease in size (Corby-Harris et al., 2019). There is a clear relationship between nutritional state, fat body size and the functionality of the HPGs, though the role of dietary lipid on expression of circulating ecdysterone hormones has not yet been elucidated.

The proportion of essential fatty acids in pollen varies with pollen source (Manning, 2001) but also has profound effects on honeybee cognition which directly affects foraging behaviour (Arien et al., 2015, 2018). Here, we have tested one dietary lipid source with a fixed proportion of the essential fatty acids (soy lecithin, 8:1 linoleic: α-linolenic acid); a ratio exceeding that known to cause impaired cognition with young honeybees. Although high dietary omega 6 fatty acids may induce physiological mechanisms to extend lifespan (O'Rourke et al., 2013; Chen et al., 2019), this has not been tested in honeybees. Owing to the potential physiological impacts of essential fatty acid imbalance, it is possible that the ratio of the essential fatty acids also influences the regulation of EEA:L.

Wright et al. (2018) predict that fat is the most variable component of the diet of adult worker honeybees, because it is also the most variable component of royal jelly. We have shown that broodless adult honeybees actively regulate their intake of protein and lipid. Foraging worker bees are likely to collect pollen with a narrow range of P:L ratios, but nurse bees still must consume food in the correct proportions to produce royal jelly. Future studies which incorporate nutrient balancing in whole colonies will elucidate how the P:L ratio in food influences the quality of royal jelly and other glandular secretions, and how this in turn might influence brood production and adult worker size and development.
